# GPS Telemetry Reveals a Zebra With Anthrax as Putative Cause of Death for Three Cheetahs in the Namib Desert

**DOI:** 10.3389/fvets.2021.714758

**Published:** 2021-08-20

**Authors:** Ruben Portas, Ortwin H. K. Aschenborn, Joerg Melzheimer, Manie Le Roux, Kenneth Heinrich Uiseb, Gábor Árpád Czirják, Bettina Wachter

**Affiliations:** ^1^Department of Evolutionary Ecology, Leibniz Institute for Zoo and Wildlife Research, Berlin, Germany; ^2^School of Veterinary Medicine, University of Namibia, Windhoek, Namibia; ^3^Ministry of Environment, Forestry and Tourism, Directorate of Wildlife and National Parks, Windhoek, Namibia; ^4^Ministry of Environment, Forestry and Tourism, Directorate of Scientific Services, Windhoek, Namibia; ^5^Department of Wildlife Diseases, Leibniz Institute for Zoo and Wildlife Research, Berlin, Germany

**Keywords:** *Acinonyx jubatus*, anthrax, *Bacillus anthracis*, cheetah, *Equus zebra hartmannae*, Namib Desert, mountain zebra, wildlife

## Abstract

Anthrax is a bacterial disease caused by *Bacillus anthracis* that affects wildlife, livestock and also humans in different parts of the world. It is endemic in some parts of Africa, including Namibia, with species differing in their susceptibility to the disease. Carnivores are typically less susceptible to anthrax than herbivores. Most carnivore species survive infection and have high seroprevalence against anthrax, whereas most herbivore species have low seroprevalence and typically die quickly when infected. Several reports have shown that cheetahs, unlike most other large carnivores, are susceptible to anthrax leading to a sudden death. This finding was suggested to be linked to the low genetic variability of cheetahs which might reduce an adequate immune response and thus explain such a high susceptibility to the disease. Here, we report an incidence of three free-ranging cheetahs that died within 24 h after feeding on a mountain zebra that tested positive for anthrax in the Namib Desert. We were able to reconstruct this incidence with the data recorded in the GPS (Global Positioning System) collar worn by one of the cheetahs and retrieved in the field. It is very likely that the cheetahs died from anthrax, although *Bacillus anthracis* could not be isolated from tissue and soil samples by bacterial culturing. The mountain zebra is the first described case of a wild animal that tested positive for anthrax in this arid area in southwestern of Namibia. We discuss the negative laboratory results of the cheetahs in the light of new insights of their immune system and its potential to mount a response against this bacteria.

## Introduction

Anthrax is an infectious zoonotic disease caused by the rod-shaped gram-positive bacteria *Bacillus anthracis*. It affects wildlife, livestock and humans world-wide, with southern and eastern Africa being affected on a regular basis with outbreaks (1, 2). Anthrax mortalities in African wildlife species have been reported regularly from national parks (NP) such as the Etosha NP in Namibia, Kruger NP in South Africa, Queen Elisabeth and Lake Mburo NPs in Uganda and Serengeti NP in Tanzania (1–4). Most mortalities are reported for herbivore species, including (in alphabetic order) black rhinoceros (*Diceros bicornis*), blue wildebeest (*Connochaetes taurinus*), buffalo (*Syncerus caffer*), elephant (*Loxodonta africana*), gemsbok (*Oryx gazelle*), giraffe (*Giraffa camelopardalis*), hippopotamus (*Hippopotamus amphibious*), impala (*Aepyceros melampus*), kudu (*Tragelaphus strepsiceros*), springbok (*Antidorcas marsupialis*) and zebra (*Equus burchelli*) (1, 2, 4). Species differ in susceptibility to the disease and are often affected by the bacteria during different times of year resulting in particular outbreak events (1, 3, 5).

In herbivores, anti-toxin antibody titers and seroprevalence are typically low (2, 6). It is suggested that this is due to the typical sudden death of infected herbivores preventing the development of a protective antibody response at population level (2). In the Etosha NP, seroprevalence in elephant, giraffe and black rhinoceros was 0% and in springbok 15%. In the Ngogongoro Crater in Tanzania, seroprevalence in wildebeest was 4% and of buffalo 14%, whereas in the Serengeti NP seroprevalence was higher with wildebeest having 19% and buffalo 46% seroprevalence (2, 6). The highly susceptible zebra had a seroprevalence of 0% in all three study sites (2, 6), however, a subsequent study in the Etosha NP demonstrated that zebras do survive sublethal infections and mount adaptive immunity, although this protection decreases rapidly if not boosted regularly with further sublethal infections (5).

In contrast to herbivores, only few suspected and confirmed anthrax mortalities were reported from free-ranging large carnivores. Examples are lions (*Panthera leo*) in Kruger NP (7), cheetahs (*Acinonyx jubatus*) in Etosha, Kruger, and Serengeti NPs (2, 8–10) and leopards (*Panthera pardus*) in Etosha NP (11). Carnivore species, except cheetahs, often have high antibody titers (mostly higher than 1:1,024 and up to 1:65,536) against anthrax toxin antigens (6, 9). Also seroprevalence is high, e.g., in Etosha and Serengeti lions it was 93 and 90%, respectively, in Etosha and Serengeti spotted hyenas (*Crocuta crocuta*) 100 and 87%, respectively, and in Etosha black-backed jackals (*Canis mesomelas*) 100% (2, 6). It is suggested that the high antibody titers and seroprevalence are associated with a protective antibody-mediated adaptive immune response of carnivores that are regularly exposed to anthrax by consuming infected meat (2, 6).

Cheetahs seem to be a special case. When infected, they die quickly as was reported from cheetahs fed unknowingly with anthrax contaminated meat in captivity (12, 13). However, similar to zebra, they do survive sublethal infections and mount adaptive immunity. Three of seven cheetahs (43%) in the Etosha NP showed low antibody titers (1:16–1:64) before they eventually died between 9.5 months and 20 months after sampling (9). These three cheetahs and four additional ones were radio-collared and followed until they died. The four additional ones all tested positive for *B. anthracis* and/or their spores after death (9). During an anthrax outbreak in the Jwana Game Reserve in Botswana, one of 23 cheetahs (4%) tested seropositive for anthrax, again demonstrating that cheetahs can develop antibodies against anthrax (13). Furthermore, a live spore vaccine (Sterne strain 34F2) was shown to also induce an antibody-mediated adaptive immune response in cheetahs with titers up to 1:256 (14).

Although cheetahs can mount an immune response against *B. anthracis*, infections typically lead to a rapid disease progression and death. It was suggested that this might be due to the lack of scavenging by cheetahs (15) and thus to a lack of regular opportunities to get exposed to anthrax infected carcasses (9). Infections of cheetahs might still occur when they kill an animal in the final state of the infection and ingest a sudden and high amount of bacilli (9).

Anthrax is endemic in Namibia (16) and has been studied mainly in Etosha NP since 1966 (8). Anthrax was reported sporadically in wildlife species from private farmland in Namibia (16), although none of the 115 cheetahs sampled on farmland between 1993 and 2005 were seropositive (17). Here, we report the first case of anthrax in a wildlife species in the Namib Desert in southern Namibia. We were able to reconstruct the death of a coalition of three cheetah males which fed on the carcass of an adult mountain zebra (*Equus zebra hartmannae*). This was possible because one of the cheetahs was collared with a high resolution GPS (Global Positioning System) collar that recorded the movements and behavior of the cheetahs. The mountain zebra tested positive for anthrax, whereas the three cheetahs tested negative for anthrax. We discuss this result in the light of new insights on the immune system of the cheetah.

## Materials and Methods

### Immobilization and GPS Collaring Methods

On the 15th of May 2019, two cheetahs of a coalition of three males were captured in box traps in the central Namib-Naukluft National Park (NKNP). They were estimated to be between 6 and 7 years old and weighted 42.5 and 45.0 kg, respectively. The two cheetahs were immobilized with a mixture of ketamine (3.0 mg/kg; Kyron Laboratories, South Africa) and medetomidine (0.06 mg/kg; Kyron Laboratories, South Africa), and reversed with atipamezole (Antisedan, 0.25 mg/kg; Zoetis, South Africa). One of the captured males was fitted with a GPS collar (e-obs GmbH, Grünwald, Germany) that recorded the positions of the cheetah every 15 min when the animal was moving and every 6 h when the animal was resting, i.e., not moving. GPS data were downloaded from the collar every 2 months during aerial tracking flights as described in Melzheimer et al. (18). Cheetah males of a coalition remain together (18), therefore the movements of one coalition partner represent those of the entire coalition. The GPS collar was equipped with a sensor that recorded the acceleration (ACC) of the movements and thus provided additional information on the activity of the animal. Such ACC data was set to be recorded every 2 min for 3.6 s. ACC devices help, for example to differentiate activity periods from resting periods (or death) at a GPS cluster, with activity signals at such cluster likely representing feeding events (19). The ACC data were examined using the program Firetail (20).

### Tissue Sampling and Bacteriological Analysis

Tissue samples from skin, nose, tongue and eye as well as buccal and nasal swabs were taken on the 27th of October 2019 from each of the dead cheetahs. Additionally, soil samples below the three carcasses were collected. A buccal and a nasal swab were taken from the dead mountain zebra, as well as soil below the carcass collected. Samples were collected following the recommendations of the EWDA Wildlife Disease Network (21). The samples were brought to the Namibian Central Veterinary Laboratory (CVL) in Windhoek for anthrax testing by bacterial culturing as described in (1). On 29th of April 2020, another set of samples containing pieces of skin and smears from the skulls and jaws from the cheetahs as well as 12 soil samples below the carcasses were taken and brought to the CVL in Windhoek and the laboratory of the Etosha Ecological Institute (EEI), again for anthrax testing by bacterial culturing.

## Results

**Figure 1:**
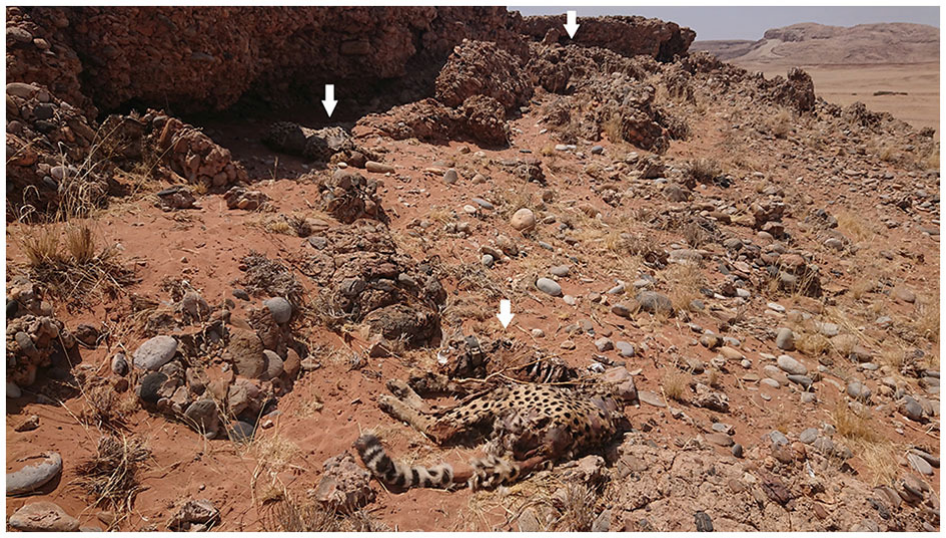
Two of the three cheetah male carcasses (left and middle arrow) as found 11 days after they died. The carcass of the third animal was found approximately 10 meters away behind a rock (right arrow).

**Figure 2:**
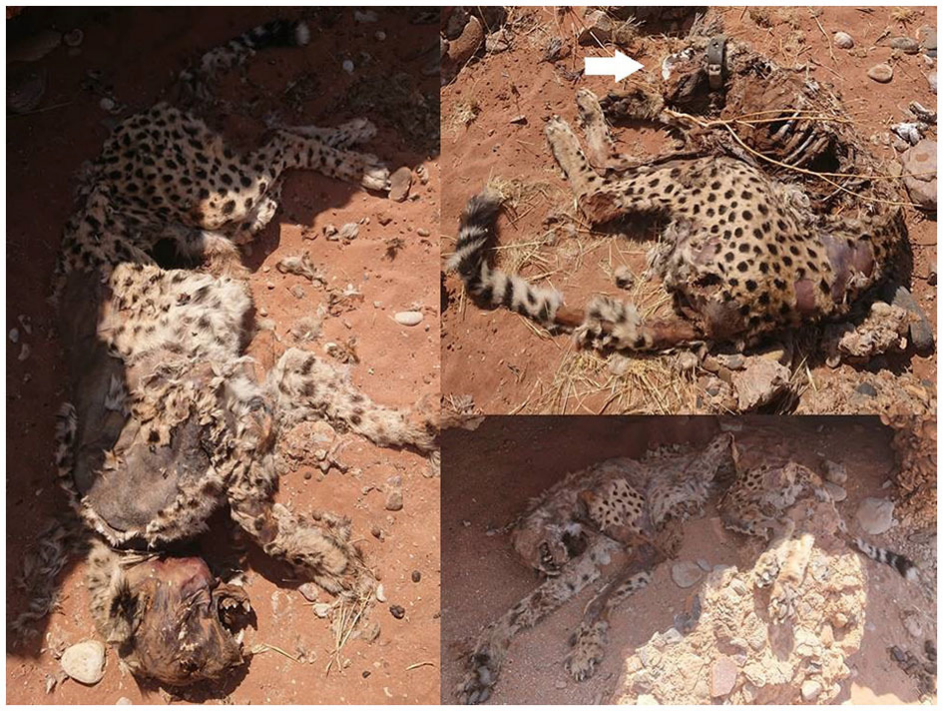
Close up of the carcasses of the three cheetahs. These pictures were taken during the second visit, 22 days after the estimated time of death. Note the GPS collar on the neck of the upper right cheetah pointed out by a white arrow.

On the 5th of October 2019, the carcass of the GPS collared cheetah was located during a radio tracking flight and seen from the aircraft. During the subsequent ground inspection, three cheetahs were found dead and within 3–10 meters from each other (Figures 1, 2). The GPS data indicated that they died on the 24th of September 2019, between 18:00 and 24:00. Due to the unexpected situation, samples were only collected during subsequent visit(s) after inspection of the data from the GPS collar of the cheetah to find any unusual GPS patterns.

**Figure 3:**
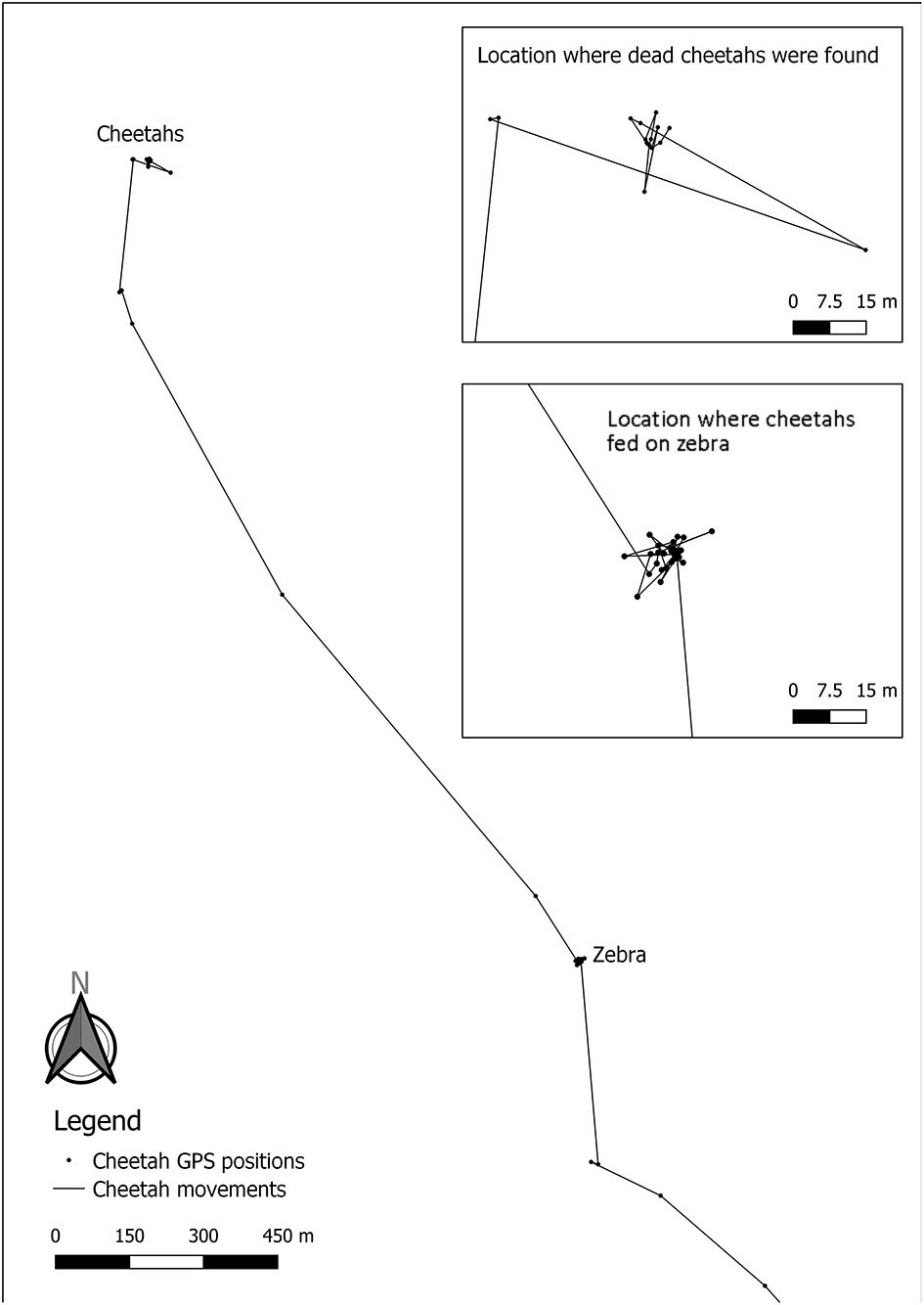
Movements of the GPS collared cheetah 2 days prior to his death. The upper insert shows the GPS clusters of the cheetah the day he died (positions taken every 6 h), while the location where cheetahs fed on zebra shows the GPS cluster at the dead mountain zebra (positions taken every 15 min). The accuracy of the GPS collar is ~6 meters.

**Figure 4:**
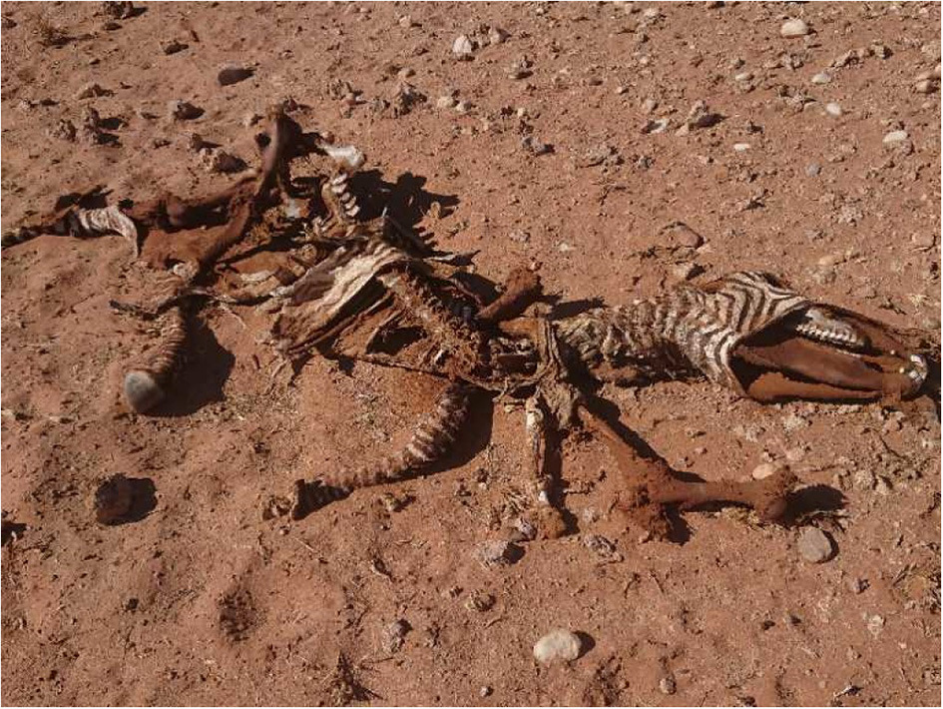
Carcass of the anthrax positive mountain zebra which the cheetahs fed on <24 h prior to their death. The picture was taken during the first field inspection, 11 days after the estimated cheetah death.

Analysis of the GPS data revealed two clusters of GPS locations. One was the place of death of the cheetahs and the other was located 1.9 km away from the location of the dead cheetahs (Figure 3). When visiting the yet unknown cluster in the field on the 27th of October 2019, a carcass of an adult mountain zebra was found (Figure 4). The cheetahs were at the zebra carcass from 08:00 on the 23rd of September 2019 to 03:30 on the 24th of September 2019, i.e., 19.5 h. The GPS collar recorded activity data within the cluster of GPS locations from 08:00 until 12:00, thus the collared cheetah was most likely feeding, then he rested until 17:45, was active again until 19:15, rested until 02:30 in the next morning and then was active again until 03:30. After the feeding event, the three cheetahs traveled from the cluster where the mountain zebra carcass was found to the cluster where their carcasses were found. The GPS data recorded disclosed gaps of 6 h suggesting that the cheetahs died between 14.5 and 20.5 h after they left the carcass. Analysis of the ACC data did not suggest that the cheetahs killed the zebra since there was no high activity over a few 100 meters, i.e., a hunting distance shortly before the cheetah settled at the carcass.

*B. anthracis* was isolated from the buccal and the nasal swabs collected from the dead zebra. This is the first reported case of anthrax infection in the NKNP and of a wildlife species in the Namib Desert. All the cheetah and soil samples tested were negative.

## Discussion

Our study is the first report on an anthrax case in the NKNP and the second case in the Namib Desert. The first case in the Namib Desert was on the 26th of November 2008, when the CVL confirmed two sheep to have died from anthrax on a farm bordering to the NKNP and 40 km east of the mountain zebra carcass in our study.

Using the GPS and ACC data of the collared cheetah, we conclude that the three cheetahs died within <24 h after having spent 19.5 h at an anthrax positive mountain zebra from which they most likely fed from. Although all samples collected from the cheetahs and the soil below their carcasses were negative for *B. anthracis*, it is highly likely that they died from anthrax. We suspect that the ingestion of zebra meat contaminated with *B. anthracis* and/or the toxin secreted by the bacteria was the cause of death of the three cheetahs. We do not know whether (a) the three cheetahs found the carcass of the zebra and scavenged from it, (b) the two non-collared cheetahs killed the zebra and the collared one joined for feeding, (c) the cheetahs killed the zebra without a hunt shortly before it died from the anthrax infection or (d) the ACC schedule was not dense enough to record a hunt. Cheetahs generally do not scavenge and only a few cases of scavenging or kleptoparasitism were reported (22, 23), but in an area with low prey availability such as in the Namib Desert, cheetah might scavenge occasionally. An alternative, although unlikely explanation for the death of the zebra and/or death of the cheetahs might be poison. The described incidence occurred in a remote area of the NKNP with restricted access to people and with no carnivore-livestock conflict or game-livestock competition for food in a distance of at least 50 km from the area.

Previous observations have shown that bacterial cultures from highly susceptible animals that die quickly, such as cheetahs, are often anthrax negative by bacterial culturing as they die at low bacteremia (9). This could explain why the microbiological analysis yielded negative results. In addition, sporulation only occurs when *anthrax bacilli*, the vegetative form, is exposed to air, thus when a carcass is opened, e.g., by scavengers (7). The exposure to air has to happen relatively quick, i.e., before the blood of the carcass becomes diluted, dispersed or desiccated, because *anthrax bacilli* need protein from the blood to prevent lysis (1, 7). When we found the carcasses of the three cheetahs 11 days after their death, they seemed untouched and not opened by scavengers, rather decomposed by insects, and their flesh and skin desiccated from the heat (Figure 2). This could further explain the negative results of the laboratory tests. The high susceptibility of cheetahs to anthrax, their low bacteremia and sudden death after infection [usually within 24–36 h (24)] may also have contributed to the low antibody titers and the low seroprevalence in previous study (9, 13), since it takes ~2 weeks to develop antibodies against a pathogen (25).

Cheetahs exhibit a low genetic variability, also at the immune genes of the major histocompatibility complex (MHC) which is responsible for an effective immune response. Although the MHC variability is not as low as originally thought, it is still lower compared to that of other carnivore species (26). It was therefore suggested that the relatively low MHC variability might be an additional factor for the high susceptibility of cheetahs to anthrax (9). However, several studies demonstrated that free-ranging cheetahs are not generally impacted by infectious diseases (27–29). Their low antibody-mediated adaptive immune response against anthrax is, though, in line with results that cheetahs have low immunoglobulin G concentrations (the predominant antibody isotope in mammals) compared to leopards, which have a high MHC variability (29). However, to compensate for this low adaptive immune potential, cheetahs have a higher constitutive innate immunity than other carnivore species, including black-backed jackals, brown hyenas (*Hyena brunnea*), caracals (*Caracal caracal*), leopards and lions (28, 29). The constitutive innate immune effectors provide a rapid first line of defense against intruders with the complement and other antibacterial proteins (e.g., lysozyme) destroying the bacterial cell walls, especially of gram-positive bacteria such as *B. anthracis* (30). Thus, cheetahs can mount an adequate response against bacteria with their potent constitutive innate immunity. However, when a high load of *B. anthracis* has been ingested from anthrax infected animals at the terminal state, their constitutive innate immunity might be overloaded and/or might destroy the bacteria with the consequence that high levels the endotoxin are passively released into the circulation, resulting to a sudden death in both scenarios (31). Other carnivore species that are less susceptible to anthrax than cheetahs may also become ill and eventually die if the dose of *B. anthracis* ingested is high (7). In these cases, typical lesions such as oedema, hemorrhages and necroses are localized in the throat, tongue, neck, stomach and intestine, body parts that preferably should be targeted for sampling (21).

A better understanding of the ecology of anthrax and its prevalence in the arid environments of Namibia is required to assess whether undetected cases in this ecosystem occur. This includes the sero-surveillance of predators and scavengers with large home ranges that might provide a sensitive indicator of anthrax distribution. In addition, such studies could provide further information on the impact that anthrax has on this cheetah population. Our case also demonstrates the utility of GPS collars ad ACC devices in wildlife disease research, especially in remote areas.

## Data Availability Statement

The original contributions presented in the study are included in the article/supplementary material, further inquiries can be directed to the corresponding author/s.

## Ethics Statement

All animal procedures were approved by the Internal Ethics Committee of the Leibniz Institute for Zoo and Wildlife Research (Leibniz-IZW, permit number 2002-04-01) and authorized by the Namibian Commission of Research, Science and Technology (authorization numbers 2018050101 and AN20191120).

## Author Contributions

RP and BW designed the study. RP and OA collected the samples. RP and JM analyzed the movement data. RP and BW wrote the first draft and GC revised it. JM, OA, ML, and KU edited the draft versions and read and approved the final manuscript. All authors contributed to the article and approved the submitted version.

## Conflict of Interest

The authors declare that the research was conducted in the absence of any commercial or financial relationships that could be construed as a potential conflict of interest.

## Publisher's Note

All claims expressed in this article are solely those of the authors and do not necessarily represent those of their affiliated organizations, or those of the publisher, the editors and the reviewers. Any product that may be evaluated in this article, or claim that may be made by its manufacturer, is not guaranteed or endorsed by the publisher.
